# Mechanoinflammation: mechanosensing and inflammatory amplification through the airway macrophage Piezo1–NLRP3 axis in cough-variant asthma

**DOI:** 10.3389/fimmu.2026.1915398

**Published:** 2026-10-08

**Authors:** Suping Yu, Xianqing Ren, Ying Ding, Yan Xu, Mengmeng Sun, Guichun Zhang, Bingxue Zhang, Qiongqiong Xing, Guihua Song, Haitao Wang

**Affiliations:** 1 Pediatric Hospital, The First Affiliated Hospital of Henan University of Chinese Medicine, Zhengzhou, Henan, China; 2 College of Pediatrics, Henan University of Chinese Medicine, Zhengzhou, Henan, China

**Keywords:** airway macrophage, chronic cough, cough hypersensitivity syndrome, cough-variant asthma, interleukin-1β, mechanotransduction, NLRP3 inflammasome, Piezo1

## Abstract

Cough-variant asthma (CVA) is characterized by chronic dry cough as its predominant manifestation, lacking the wheezing typical of classic asthma. Violent paroxysmal coughing generates distinct mechanical loads across different airway segments: the larynx and glottis participate in airflow generation; the intrathoracic trachea and main bronchi undergo dynamic compression; bronchial bifurcations and narrowed segments experience elevated wall shear; and distal lung tissue is mainly subjected to volume change and tissue stretch. To address this gap, this review introduces “mechanoinflammation” as a concept—a pathogenic process in which physical forces directly gate mechanosensitive ion channels on immune and structural cells to initiate sterile innate immune cascades and establish self-amplifying inflammatory loops, distinct from the existing concept of “mechanotransduction,” which focuses on physiological force-to-signal conversion. Alveolar, interstitial, and inflammation-recruited macrophages occupying different niches may sense corresponding local mechanical changes and together constitute the candidate cellular basis of airway macrophage mechanotransduction. Cough-induced membrane tension may open Piezo1 and mediate calcium influx, which is amplified through the PI3K/AKT axis to license NLRP3 inflammasome assembly and mature IL-1β release. The latter may sensitize vagal C-fiber terminals and may close a physical–immune–neural feedback loop in which coughing initiates inflammation and inflammation reinforces coughing. This signaling axis provides a possible mechanistic explanation for corticosteroid resistance in refractory CVA and provides novel therapeutic directions targeting Piezo1 modulators, NLRP3 inhibitors, and IL-1 blockade to disrupt chronic cough.

## 1 Introduction

Chronic cough is among the most common symptoms prompting patients to seek medical care; persistent, intractable cough not only substantially impairs quality of life but also imposes a heavy burden on healthcare resources. Etiologically, cough-variant asthma (CVA) is one of the most important causes of chronic cough in both adults and children, estimated to account for 25% to 42% of chronic cough cases across different populations, and it has long been underdiagnosed and subject to diagnostic delay because of its insidious onset, lack of specific signs, and limitations of objective diagnostic testing (1, 13). In China, CVA has been listed by multiple cough management guidelines as the leading cause of chronic cough, and its standardized diagnosis and treatment have become an important topic in respiratory medicine and allergy (12). The core clinical feature of CVA is chronic dry cough as the sole or predominant manifestation, notably lacking the wheezing and dyspnea typical of classic asthma; yet its airway pathology is highly homologous to that of classic asthma: eosinophil counts in bronchoalveolar lavage fluid and airway tissue, as well as serum eosinophil cationic protein levels, are all markedly elevated, indicating that it is fundamentally a type 2, eosinophilic airway inflammatory disease (4, 6). The reason patients cough without wheezing is, in part, that their wheezing threshold is higher than in classic asthma—that is, more severe airway obstruction is required to produce audible wheeze (5).

CVA should not be seen as a static or harmless condition. Multiple follow-up studies have shown that a substantial proportion of patients progress to classic asthma within several years; in pediatric cohorts this conversion rate can reach approximately 50%, and age at onset—rather than the severity of airway hyperresponsiveness—is the key risk factor predicting conversion (2). Large-sample induced-sputum studies have further identified CVA as an early stage of classic asthma and revealed internal inflammatory heterogeneity, including eosinophilic and non-eosinophilic phenotypes, indicating that eosinophilic inflammation alone does not suffice to encompass all of its pathogenic mechanisms (14). Correspondingly, early, standardized anti-inflammatory intervention has been shown to significantly suppress the development of wheezing, suggesting that timely interruption of airway inflammation may meaningfully alter the natural course of the disease (3). For this reason, inhaled corticosteroids (ICS) are unanimously recommended by guidelines across countries as the first-line treatment for CVA, with leukotriene receptor antagonists as a supplement (8, 9). However, in clinical practice, outcomes often fall short: a considerable proportion of patients respond poorly to ICS; the longer the duration of chronic cough and the greater the sensitivity to cold-air stimulation, the worse the short-term efficacy of corticosteroids, and some patients even require prolonged courses or the addition of other agents to achieve control (7). Frameworks centered solely on type-2 chemical inflammation remain insufficient for all patients. This insufficiency is particularly evident in subgroups with refractory CVA, non-type-2/mixed-granulocytic phenotypes, or cough-hypersensitivity-dominant presentations showing poor responsiveness to ICS, where innate immune and physical inflammatory mechanisms require urgent elucidation.

In fact, the unifying mechanism of the chronic-cough phenotype is increasingly attributed to heightened excitability of the cough reflex pathway at both peripheral and central levels, a condition termed cough hypersensitivity syndrome. This paradigm emphasizes that patients can mount an excessive cough response to low-level thermal, chemical, and even mechanical stimuli (10, 11). The paradigm has been further applied to understanding chronic refractory cough and has provided a conceptual framework for exploring its novel treatments (15). Notably, in classic formulations of cough hypersensitivity syndrome, mechanical stimulation is explicitly listed as one of the low-threshold factors capable of triggering intractable cough, yet its downstream molecular transduction mechanism has long remained vague. This highlights a fundamental biophysical fact overlooked by the chemically centered immunological model: the act of coughing is itself a violent mechanical event. During a typical paroxysm, the larynx and glottis of the upper airway participate in airflow generation; after glottal opening, rapid expiration propagates through the lower airway. Intrathoracic airways downstream of the equal-pressure point undergo dynamic compression, and endoscopic evidence specifically shows that the trachea and main bronchi can collapse markedly during coughing (19, 20); computational fluid dynamics studies further indicate that wall shear at small-airway bifurcations and regions of heterogeneous narrowing exhibits marked spatial heterogeneity (22, 23), showing that cough-generated mechanical load is differentially distributed across airway anatomical segments. More importantly, the mechanical stress imposed by long-term, repeated severe coughing is recognized as one of the independent factors driving airway remodeling (13). This gives rise to a core question that has long been evaded: in CVA—a disease whose sole prominent manifestation is cough—is the purely physical force generated by coughing itself an independent and powerful proinflammatory driver?

Recent advances in innate immune mechanobiology provide important molecular evidence to address this question. Studies have confirmed that hydrostatic pressure cycling, similar to that experienced by immune cells within the lung, can directly initiate the inflammatory response of innate immune cells via the mechanosensitive ion channel Piezo1, and that conditional deletion of Piezo1 specifically in innate immune cells markedly attenuates pulmonary inflammation in the settings of bacterial infection or fibrosis-associated autoinflammation (16). On this basis, the molecular bridge between mechanical force and classic innate immune effectors has been progressively revealed: Piezo1-mediated mechanical gating can directly drive activation of the NLRP3 inflammasome through calcium influx and calcium-activated potassium channels, and shear stress alone is sufficient to serve as the second signal required for NLRP3 activation, eliciting gasdermin D cleavage and IL-1β secretion (17, 73). The Piezo channel family is thus recognized as an integrative hub for mechanical and immune signaling, exerting gatekeeping functions in inflammation, pain, and neuroimmune interaction (18). Together, these findings suggest that the mechanical force–Piezo1–inflammasome axis is a plausible key link connecting coughing—a macroscopic physical event—with microscopic airway inflammation and may also represent the molecular mechanism by which mechanical stimulation in cough hypersensitivity syndrome provokes cough. Given that this process encompasses a complete pathological chain from physical force sensing to innate immune amplification to self-sustaining inflammatory loops—transcending the existing concept of “mechanotransduction,” which focuses on physiological force-to-signal conversion—this review collectively terms it “mechanoinflammation.” This term has recently been adopted in the mechano-immunology field (18).

Building on this background, this review aims to systematically dissect the molecular mechanisms of airway macrophage mechanosensing in CVA and, accordingly, to propose a paradigm shift from chemical immunity toward mechanoinflammation. Lung macrophages exhibit marked spatial heterogeneity: alveolar macrophages are distributed on the airspace surface, interstitial lung macrophages occupy tissue niches such as the lung parenchyma and bronchovascular bundles, and inflammation-recruited macrophages may enter lung tissue or the airspace (107). The central hypothesis of this work is that alveolar, interstitial, and inflammation-recruited macrophages in different niches may sense corresponding lower-airway mechanical changes and, through surface Piezo1 channels and the Piezo1–Ca²^+^–PI3K/AKT–NLRP3 signaling cascade, translate local mechanical load into inflammatory cytokine release, ultimately sensitizing local vagal sensory nerves and forming a self-reinforcing closed loop in which coughing initiates inflammation and inflammation, in turn, reinforces coughing (**Figure 1**). This hypothesis is developed stepwise across biofluid mechanics, channel structural biology, intracellular signaling cascades, neuroimmune coupling, and therapeutic translation, with the aim of assigning concrete molecular substance to the long-vague concept of “mechanical stimulation” in cough hypersensitivity syndrome and providing a new explanatory dimension for corticosteroid resistance in a subset of patients.

**Figure 1:**
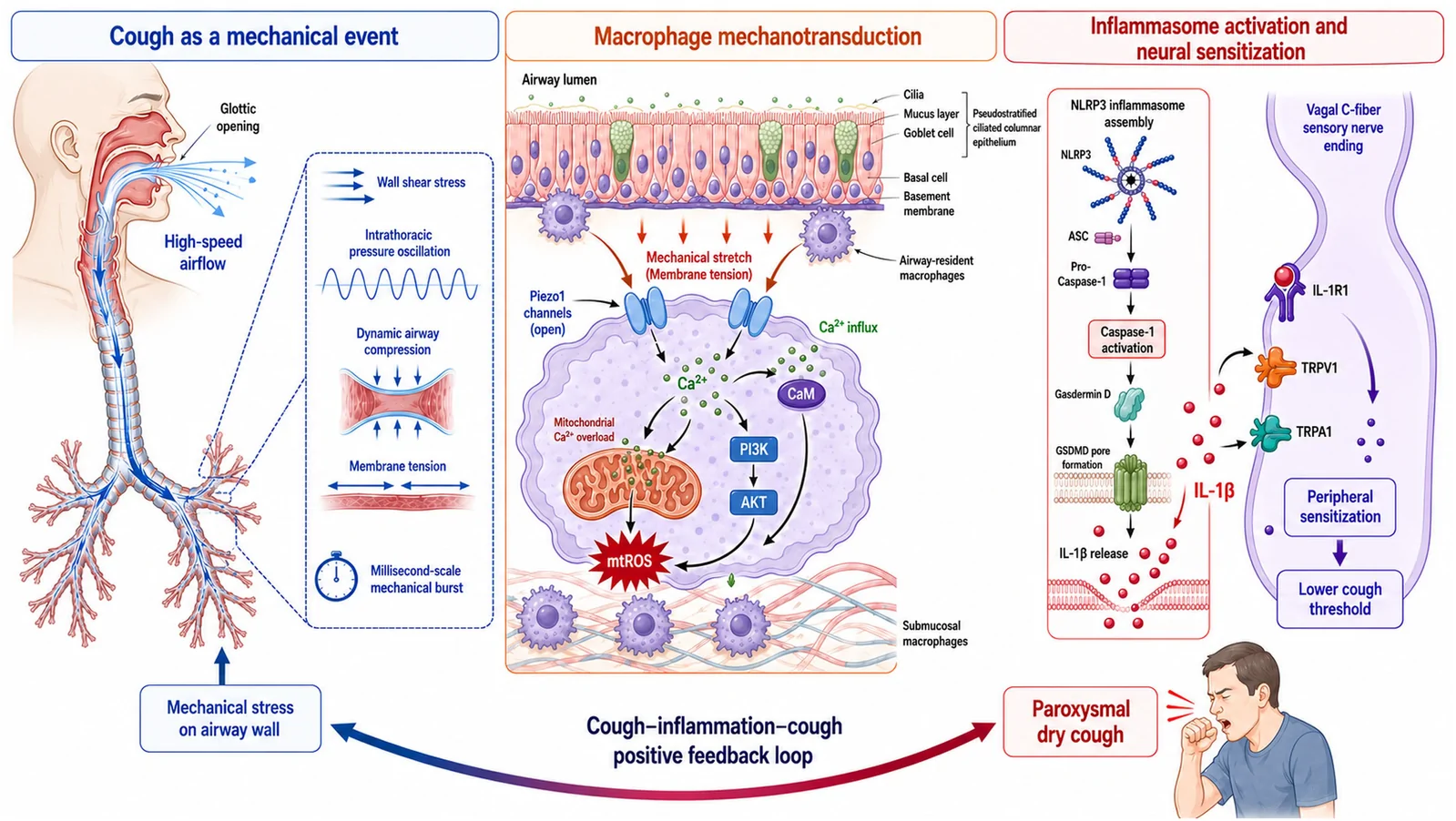
Hypothetical overall mechanism by which cough-generated mechanical force connects inflammation and neural hypersensitivity through a candidate airway macrophage Piezo1–NLRP3 pathway. Left: upper-airway glottal opening and region-specific lower-airway loads, including dynamic compression of the intrathoracic trachea and main bronchi, wall shear at bronchial bifurcations and narrowed segments, and volume change with tissue stretch in distal lung tissue. Center: alveolar, interstitial, and recruited macrophages receive corresponding local mechanical inputs and link Piezo1, Ca²^+^, PI3K/AKT, and mitochondrial reactive oxygen species to NLRP3 activation. Right: mature IL-1β release and vagal C-fiber sensitization; the looping arrow at the bottom denotes the cough–inflammation feedback loop.

## 2 Fluid mechanics of the cough microenvironment and tissue stress remodeling constitute the physical basis of mechanoinflammation

To establish the plausibility of mechanical stress as a pathogenic factor in CVA, one must first quantify the physical changes that coughing produces in the airways from a biofluid mechanics perspective. A violent cough is not just a simple blast of air but a series of highly coordinated, energy-dense mechanical events that, within a millisecond timescale, substantially disrupt local airway tissue homeostasis and set a physical load for resident immune cells far beyond physiological norms.

### 2.1 Violent paroxysmal coughing generates extremely high wall shear stress on the airway mucosal surface through high-velocity turbulence during the expiratory phase

An effective cough comprises, in sequence, a deep inspiration, a compression phase with the glottis tightly closed, and an explosive phase with the glottis abruptly opening. During the compression phase, forceful contraction of the expiratory muscles sharply raises alveolar and intrathoracic pressure; when the glottis opens transiently, gas rushes out at extremely high velocity driven by the enormous pressure gradient, the instantaneous Reynolds number within the airway rises substantially, and the previously orderly laminar flow is converted into intense turbulence (19). The opening dynamics of the glottis directly determine the intensity of the airflow burst; during coughing, the amplitude and velocity of vocal-fold abduction are highly coupled with peak expiratory flow and volume acceleration, so that subtle differences in glottal motion can substantially alter the airflow shear intensity acting on downstream airways (21). The velocity gradient between the high-velocity airflow and the airway mucosal surface is converted, at the wall, into wall shear stress. Airway models based on computational fluid dynamics show that, even under steady-state inspiratory flow, the wall shear stress on the carinal side of small-airway bifurcations exhibits marked spatial heterogeneity, and its peak values are far higher than those estimated by early one-dimensional approximations (22). More importantly, when non-uniform narrowing is present in the airway, airflow-related shear stress can be locally amplified many-fold, sufficient to reach stress levels that damage the airway epithelium (23). Studies using ex vivo airway-on-chip models further confirm that airflow-induced wall shear stress can directly regulate the morphology, mucus secretion, and inflammatory level of the airway epithelium (24). Given that patients with CVA already have airway hyperresponsiveness and varying degrees of luminal narrowing, the high-velocity turbulence generated by violent coughing is very likely to form wall shear stress locally on the mucosa that is markedly higher than physiological norms, thereby providing a direct mechanical input for mechanoinflammation. This shear stress first acts on the lower-airway epithelial surface and may alter the mechanical microenvironment of neighboring macrophages through epithelial deformation, tissue compression, and matrix stretch.

### 2.2 Severe oscillation of intrathoracic pressure remodels the mechanical microenvironment of the airway wall through dynamic compression downstream of the equal-pressure point

The pathogenicity of coughing arises not only from luminal-surface airflow shear but also from the structural deformation of the airway caused by severe oscillation of transmural pressure. According to the physiology of forced expiration, intraluminal pressure along the course of the airway decreases progressively from the periphery toward the airway opening; downstream of the equal-pressure point, where intraluminal pressure equals transthoracic pressure, the airway is in a state of dynamic compression. Once expiration enters the flow-limited phase, further increases in transthoracic pressure are converted mainly into compression of the airway rather than into increased flow (19). Esophageal pressure, airflow measurements, and endoscopic observation show that during forced expiration and coughing, the intrathoracic trachea and main bronchi downstream of the equal-pressure point can undergo marked dynamic collapse (20), thereby forming a lower-airway compressive load distinct from upper-airway glottal motion. Repeated compression–reexpansion may alter the biomechanical microenvironment of the intrathoracic conducting airway wall. Bronchoconstriction and compressive stress can mediate calcium signaling via mechanosensitive cation channels and are accompanied by upregulation of remodeling mediators (27); long-term cough-related mechanical stress may participate in airway remodeling (13).

The mechanical environment of the CVA airway is already altered before coughing occurs. Airway hyperresponsiveness—a hallmark of CVA—causes exaggerated smooth muscle contraction, which increases airway wall stiffness and narrows the lumen, so that resident cells experience elevated baseline tension and compressive stress (13, 108). When violent coughing is superimposed on this pre-existing mechanical state, lung macrophages in different niches may receive additional mechanical inputs of varying magnitude and alter their Piezo1 activation threshold.

### 2.3 Fluid shear force and airway-wall stretch may influence the proinflammatory state of different lung macrophage populations

Alveolar macrophages, interstitial lung macrophages, and inflammation-recruited macrophages occupy different niches (107), and their mechanical inputs may arise respectively from airspace fluid changes, tissue compression, or matrix stretch, thereby forming spatially differentiated proinflammatory responses. The lung continuously subjects its resident cells to multiscale mechanical loads, and aberrant mechanical loading is a shared feature of chronic respiratory diseases such as asthma, chronic obstructive pulmonary disease, and pulmonary fibrosis (30, 31). At the cellular level, abundant evidence indicates that mechanical force can directly reprogram the functional phenotype of macrophages. *In vivo* studies show that different fluid shear stress conditions can determine the direction of macrophage polarization—toward the proinflammatory M1 type or the regulatory M2 type—with a low-shear microenvironment tending to promote the M1 program (32). Ex vivo quantitative studies further confirm that fluid shear stress, as its intensity and duration increase, drives human monocyte-derived macrophages toward a proinflammatory phenotype and markedly upregulates the secretion of proinflammatory mediators such as tumor necrosis factor-α (TNF-α), interleukin-8 (IL-8), and interleukin-18 (IL-18) (33). Concurrently, mechanical stretch of the airway wall constitutes an independent proinflammatory input: cyclic stretch can upregulate the release of IL-8 from bronchial epithelium and rearrange the cytoskeleton via the Rho kinase and focal adhesion pathways (26), and can also alter epithelial arachidonic acid metabolism and prostaglandin synthesis through an oxidant-mediated pathway, thereby regulating airway inflammation and smooth muscle tone (25); at the whole-lung level, the strong turbulence represented by high airflow velocity amplifies the inflammatory signal of pulmonary IL-8 via the mitogen-activated protein kinase pathway (29). It should be noted that the effect of mechanical stress on the airway is intensity- and pattern-dependent: low-intensity cyclic stretch may primarily induce a myogenic response manifested as increased tension rather than marked proinflammatory activation (28). The regulation of macrophage function by mechanical stress exhibits context-dependence, with the net effect determined by the combination of signal intensity, duration, and frequency. During liver fibrosis resolution, the mechanical stimuli sensed by macrophages arise from gradual changes in matrix stiffness—low amplitude and prolonged duration—and Piezo1-mediated calcium influx is maintained at moderate levels, promoting apoptotic cell clearance (efferocytosis) and tissue repair via Rac1-dependent cytoskeletal remodeling (43); in bone tissue, Piezo1 drives M2 polarization and osteogenic differentiation via the YAP signaling axis (35). In these physiological or reparative contexts, calcium signaling is effectively buffered by cellular homeostatic mechanisms and does not trigger mitochondrial overload. In the CVA airway, the mechanical impact of coughing has the characteristics of high amplitude (transient intrathoracic pressure reaching hundreds of cmH_2_O), high frequency (paroxysmal coughing can repeat dozens of times within a short period), and instantaneous burst (millisecond scale); Piezo1-gated calcium influx far exceeds cellular buffering capacity, and the calcium signal shifts from a pro-phagocytic homeostatic regulator to a driver of mitochondrial calcium overload and mtROS bursts, redirecting the downstream pathway from the Rac1–efferocytosis axis to NLRP3 inflammasome assembly and IL-1β maturation. Taken together, the high shear and severe stretch produced by coughing may influence corresponding lung macrophage populations across different tissue niches and form spatially differentiated proinflammatory responses (**Figure 2**). The essence of the above context-dependence is a quantifiable mechanical threshold. Normal tidal breathing produces periodic stretch (5–10% strain) and low-level laminar shear that remain below the gating tension threshold of Piezo1 (approximately 1.4–5.0 mN/m) (38, 39), primarily eliciting myogenic adaptation and maintaining tissue homeostasis without triggering inflammasome assembly. Violent paroxysmal coughing imposes a mechanical load—transient intrathoracic pressure peaks reaching hundreds of cmH_2_O, wall shear stress exceeding 10–30 Pa (22, 23)—that breaches the physical activation thresholds of Piezo1 and its downstream calcium-activated potassium channel KCNN4 (KCa3.1), forcing transmembrane ion fluxes beyond cellular buffering capacity and driving mitochondrial Ca²^+^ overload and mtROS bursts to license NLRP3 inflammasome assembly. Mechanical ventilation–associated lung injury provides a direct analogy: in alveolar macrophages, cyclic mechanical stretch activates the NLRP3 inflammasome, induces mature IL-1β release, and contributes to inflammatory lung injury (103). In RAW264.7 macrophages, mechanical strain and pharmacological Piezo1 activation increase inflammatory mediator expression, whereas Piezo1 inhibition attenuates the response (104). These studies establish that mechanical strain can engage NLRP3- and Piezo1-dependent inflammatory responses in macrophages, supporting the plausibility of a mechanically gated transition; they do not, by themselves, define a universal quantitative threshold separating physiological from pathological strain. It should be emphasized that the above threshold is not a fixed constant. Electrophysiological measurements show that the half-activation tension (T_50_) of Piezo1 is approximately 1.4 mN/m in cell-attached patches but rises to 4.7 mN/m in inside-out patches (39), indicating that the cytoskeleton and membrane lipid environment exert significant modulation on channel mechanical sensitivity. In the CVA airway, airway hyperresponsiveness-induced excessive smooth muscle contraction increases airway wall stiffness and narrows the lumen, and may alter the baseline mechanical environment and effective activation threshold of different lung macrophage subsets.

**Figure 2:**
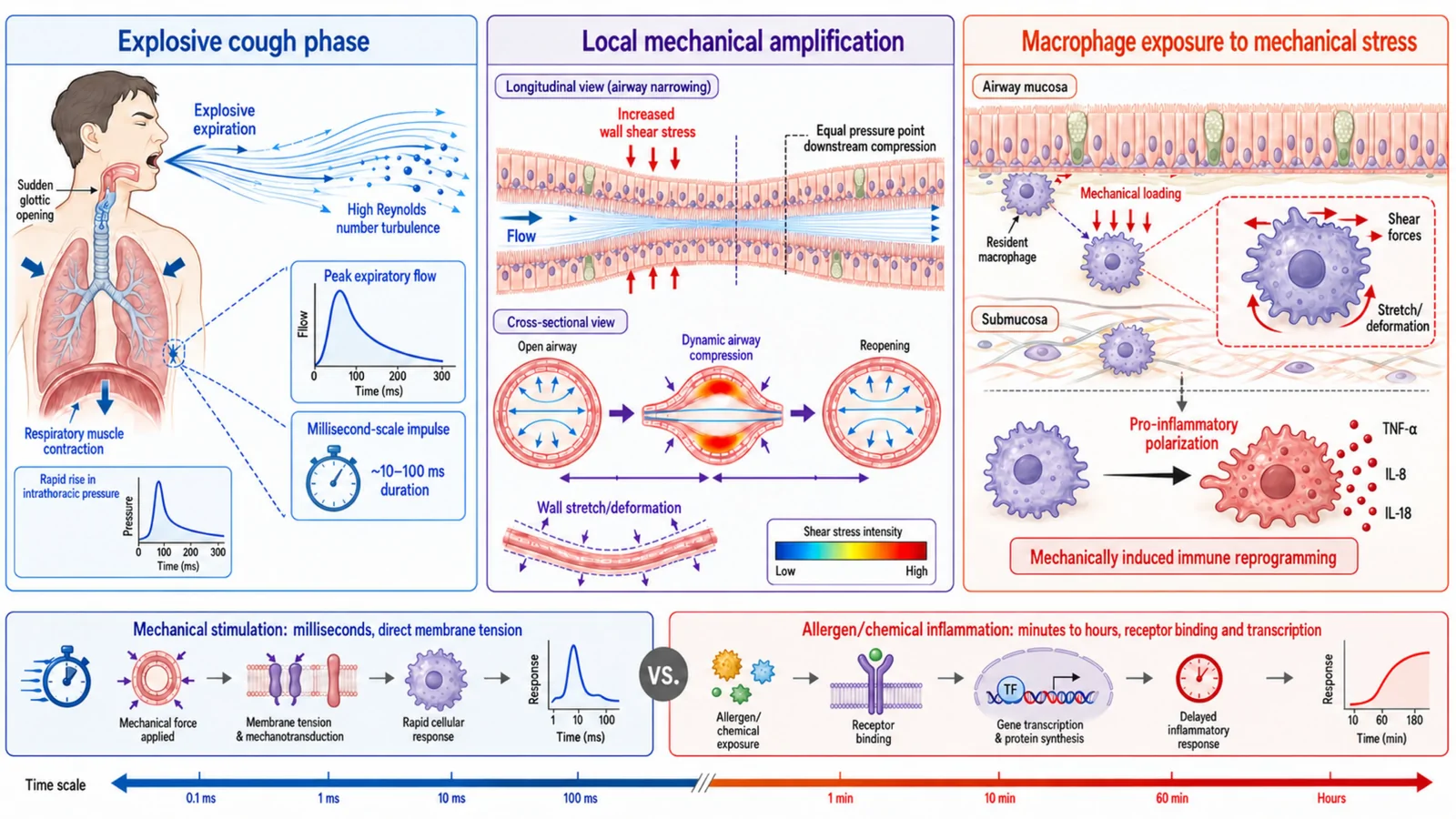
The mechanical microenvironment of coughing: turbulent airflow, wall shear stress, and dynamic airway compression. Left column (Explosive cough phase): during forceful coughing, contraction of the expiratory muscles, abrupt opening of the glottis, and rapid rise of intrathoracic pressure occur; the blue arrows denote high-Reynolds-number turbulent expiratory airflow, and the curves below depict the millisecond flow and pressure impact. Middle column (Local mechanical amplification): longitudinal and cross-sectional views show non-uniform narrowing of the airway: streamlines are densely packed at the narrowing, a high-value region of wall shear stress appears on the airway wall (the blue-to-red heat map represents shear stress intensity from low to high), accompanied by repeated compression and reexpansion of the airway wall and dynamic compression downstream of the equal-pressure point. Right column: alveolar, interstitial, and recruited macrophages in different niches may respectively receive mechanical inputs such as airspace fluid changes, tissue compression, or matrix stretch. The timeline at the bottom contrasts the kinetic difference between mechanical stimulation (millisecond, acting directly through membrane tension) and allergen/chemical inflammation (minutes to hours, dependent on receptor binding and transcription).

Alveolar type I epithelial cells express Piezo1 and can respond to mechanical stretch (36). Epithelial mechanotransduction may contribute to the inflammatory microenvironment by releasing alarmins (IL-33 and TSLP) and ATP (109, 110), and may provide a priming signal for macrophage NLRP3 activation and form a proinflammatory pathway parallel to the macrophage Piezo1–NLRP3 axis.

### 2.4 The millisecond transient burst character of cough mechanical stimulation distinguishes it from the slow kinetics of chemical drive

Mechanoinflammation can emerge as a relatively independent pathogenic pathway in CVA because it differs fundamentally from classic chemical immunity in temporal kinetics. Allergen-driven inflammation depends on slow penetration of antigen into the submucosa, binding to specific receptors, antigen presentation, and the ensuing adaptive immune activation—a series of biological steps that usually proceed over minutes to hours. In sharp contrast, the mechanical stress produced by coughing has a millisecond transient burst character: high-velocity turbulent shear and wall deformation act on the cell surface almost simultaneously with glottal opening and can directly affect mechanosensitive structures of the membrane surface without undergoing time-consuming steps such as ligand recognition. Research in innate immune mechanobiology provides direct support for this shortcut on the temporal scale—hydrostatic pressure cycling similar to that experienced by immune cells within the lung can rapidly initiate the inflammatory response in innate immune cells via mechanosensitive channels, constituting an environmental sensing mechanism independent of classic pattern recognition (16). This high-frequency, high-intensity, and near-instantaneous physical impact can bypass the time-consuming antigen-dependent steps and directly ignite an early innate immune response, explaining at the biophysical level why, in patients with CVA, seemingly minor stimuli such as cold air, speaking, and even deep breathing can provoke violent and uncontrollable paroxysmal dry cough. This transient character also implies that effective intervention must target the most upstream link of mechanotransduction rather than rely on modulating slow chemical processes.

## 3 Piezo1 as a candidate precision transducer by which airway macrophages translate mechanical force into ionic signals

The key linking macroscopic physical stress to microscopic biological signals is whether the cell surface possesses a molecular apparatus capable of directly sensing membrane deformation and converting it into transmembrane ionic flux. The mechanosensitive ion channel Piezo1 is considered the core molecule of this transduction process. Studies of pulmonary myeloid cells and macrophages of different origins support a role for Piezo1 in mechanosensing and calcium influx, with expression level and subcellular localization varying across cellular niches.

### 3.1 Piezo1 embeds in the plasma membrane in a three-bladed propeller conformation and achieves ligand-independent, purely mechanical gating through membrane tension

Piezo1 is a giant homotrimeric transmembrane channel composed of more than 2,500 amino acid residues. High-resolution cryo-electron microscopy structures reveal that its detergent-solubilized complex adopts a three-bladed propeller shape, with each protomer containing a curved transmembrane region composed of at least 26 transmembrane helices, formed by a series of concatenated four-transmembrane-helix units that constitute flexible blades; the central ion pore is enclosed by the carboxy-terminal domain, and the peripheral blades are connected to the central pore region through a tilted beam and anchor domain, providing the structural basis for allosteric gating regulation (37). This unique conformation determines a working mode for Piezo1 entirely distinct from that of traditional ligand-gated or voltage-gated channels. The structure-based membrane dome model indicates that the blades of Piezo1 locally curve the surrounding lipid bilayer into a dome-shaped depression; when the plasma membrane is subjected to tension, the dome tends to flatten and its projected area in the membrane plane increases, and the gating energy changes with the projected area, so that the channel is regulated by membrane tension with extremely high sensitivity (38). Electrophysiological studies further confirm that the sensitivity of Piezo1 in responding to lateral membrane tension far exceeds that of other mechanosensitive channels, and that resting membrane tension can tune its overall mechanosensitivity by driving channel inactivation, enabling it to work efficiently and adaptively in diverse cellular environments (39). This purely mechanical gating property—taking membrane tension as the sole sufficient stimulus and independent of any chemical ligand—has been systematically summarized as the curvature-based gating paradigm of the Piezo family (40). This property makes Piezo1 a preferred candidate molecule for directly converting the cough-induced membrane deformation into ionic signals, without undergoing any time-consuming chemical recognition step. The quantitative relationship between membrane tension and gating energy establishes the physical foundation for converting force into ionic signals: single-channel electrophysiological measurements demonstrate that the activation tension threshold for Piezo1 mechanical gating ranges from ~1.4 to 5.0 mN/m (38, 39); membrane tension fluctuations generated by cough-induced shear stress and wall strain fall well within and breach this gating threshold, triggering channel opening and mediating large-scale Ca²^+^ influx.

### 3.2 Studies of pulmonary myeloid cells and macrophages of different origins support Piezo1 mechanosensing capacity

Functional expression of Piezo1 in innate immune cells is a prerequisite for its participation in airway mechanoinflammation. Studies confirm that pulmonary myeloid innate immune cells highly express Piezo1 and can sense, via this channel, hydrostatic pressure cycling similar to that of the intrapulmonary environment to initiate an inflammatory response (16). In macrophages, Piezo1 is not only abundantly expressed but is the core mechanosensor for detecting microenvironmental stiffness, determining the magnitude of calcium influx and the direction of polarization (34). Matrix stiffness can further drive macrophage polarization toward the proinflammatory M1 phenotype via the Piezo1–Yes-associated protein (YAP) signaling axis, whereas inhibiting the downstream effector YAP can promote their repolarization to the anti-inflammatory M2 phenotype—providing a transcriptional pathway linking mechanical signals to macrophage inflammatory fate via Piezo1 (35). Studies in alveolar type I epithelial cells show that Piezo1 can localize to caveolae and respond to stretch (36), suggesting that different cells in the alveolar region may complete mechanosensing through their respective membrane structures. Airway epithelial cells, smooth muscle cells, and sensory neurons also express mechanosensitive channels and respond to physical stress (36, 78, 111). The central place of airway-resident macrophages in this framework stems from their combined anatomical sentinel position and their biochemical property as a major local source of NLRP3-dependent mature IL-1β: they constitute a relay that translates macroscopic physical load into paracrine cytokines and sensitizes nerve terminals. Piezo1 expression, membrane-domain localization, and mechanical thresholds of different lung macrophage subsets may vary with niche and activation state. In addition to Piezo1, airway macrophages express several other mechanosensitive channels, including TRPV4 (112), as well as potentially mechanoregulatory TRPA1 and the two-pore-domain potassium channel TREK-1. TRPV4 functions as a calcium-permeable cation channel that acts synergistically with Piezo1: initial Ca²^+^ influx and cytoskeletal rearrangement triggered by rapid Piezo1 gating lower the activation threshold of TRPV4, sustaining intracellular Ca²^+^ signaling (113); The coupling between Piezo1 and TRPV4 has a clear molecular basis: Piezo1-mediated calcium influx can promote arachidonic acid release through phospholipase A2 (PLA2)–related pathways, and arachidonic acid and its metabolites (such as 5,6-EET) directly gate TRPV4 open, thereby converting the rapid mechanical response of Piezo1 into TRPV4-mediated sustained calcium signaling (113, 114). The functional consequences of TRPV4 in macrophages are context-dependent: on one hand, TRPV4 promotes macrophage phagocytosis and basal defense functions (115), and in alveolar macrophages TRPV4 deficiency paradoxically increases IL-1β and CXCL1 secretion, suggesting that under certain conditions it exerts anti-inflammatory effects by limiting NF-κB signaling (116); on the other hand, excessive TRPV4 activation drives M1 polarization and proinflammatory cytokine release via the ROS/NLRP3 pathway (117). In the specific context of high-frequency violent mechanical stress in the CVA airway, TRPV4 may be driven by persistent Piezo1 activation toward a proinflammatory direction, although this inference remains to be directly validated in airway macrophages. Conversely, TREK-1 mediates K^+^ efflux under mechanical stretch to induce membrane hyperpolarization (118), and in some cellular models serves as a negative feedback mechanism to buffer excessive mechanosensitivity. These mechanosensitive channels may jointly participate in macrophage sensing and regulation of airway physical stress.

### 3.3 Mechanical opening of Piezo1 triggers rapid influx of calcium ions down their electrochemical gradient and disrupts macrophage ionic homeostasis

Piezo1 is a nonselective cation channel with good permeability to calcium ions. Because a difference of several orders of magnitude in calcium ion concentration exists between the extracellular fluid and the cytoplasm, and the resting membrane potential provides an inward electrical driving force, once Piezo1 is gated open under membrane tension, extracellular calcium ions rapidly flood into the cytoplasm down the strong transmembrane electrochemical gradient. Using genetically encoded calcium reporter systems, researchers have directly demonstrated that calcium influx in macrophages depends on Piezo1 and increases with matrix stiffness, and that this calcium signal is coupled to proinflammatory activation of macrophages (34). This membrane-tension-driven calcium influx has rapid kinetics on a microsecond-to-millisecond scale and, almost simultaneously with the mechanical stimulus, completely disrupts the calcium homeostasis of macrophages in the resting state. Given that the calcium ion is one of the most important intracellular second messengers, this physical calcium pulse may constitute the upstream initiating signal of the mechanoinflammatory cascade: it is both the starting point for activation of the calmodulin-dependent kinase network and a key upstream input for mitochondrial calcium uptake and NLRP3 inflammasome activation. Thus, the mechanical opening of Piezo1 converts a purely physical event—an increase in membrane tension—into a clear biochemical initiating signal, namely a sharp rise in cytoplasmic free calcium concentration, completing the critical transition from physical force to biochemical signal.

### 3.4 Multi-tissue genetic evidence suggests that macrophage Piezo1 may serve as a causal driver of calcium-dependent inflammation rather than a bystander molecule

Establishing Piezo1 as a core candidate molecule of airway mechanoinflammation requires causal evidence beyond correlation, and recent myeloid-specific genetic manipulation studies spanning multiple organ systems provide important support. Knockout of Piezo1 in macrophages can markedly attenuate inflammation and promote injury repair, while the positive feedback between Piezo1 and actin further amplifies macrophage activation, indicating that this channel occupies an important position in mechanoinflammation (34). In a liver fibrosis model, myeloid-specific Piezo1 knockout markedly reduces macrophage infiltration, suppresses M1 polarization, and downregulates proinflammatory cytokines, with the mechanism involving Piezo1-mediated calcium influx activating calcium-dependent proteases and thereby regulating downstream inflammatory programs (42). In a chronic inflammatory bowel disease model, macrophage Piezo1 deletion can suppress the expression of NLRP3 and nuclear factor-κB and the nuclear translocation of p65 in colonic tissue and bone marrow-derived macrophages, whereas activating macrophage Piezo1 enhances proinflammatory cytokine secretion via the NLRP3/nuclear factor-κB pathway—the first *in vivo* evidence linking Piezo1 to the NLRP3 pathway (44). In the lung, conditional knockout of Piezo1 specifically in innate immune cells can abolish pulmonary inflammation in the settings of bacterial infection or fibrosis-associated autoinflammation, demonstrating the key role of Piezo1 in pulmonary mechanoinflammation (16). It should be noted that the role of Piezo1 as a mechanosensor is broad and context-dependent, exhibiting pro-inflammatory or pro-resolving roles in different pathological backgrounds—for instance, enhancing macrophage efferocytosis to promote liver fibrosis resolution (43). Piezo1-mediated efferocytosis and pro-inflammatory signaling correspond to distinct mechanical stimulation modes: under baseline matrix stiffness or moderate mechanical cues, Piezo1 activation promotes apoptotic cell clearance and tissue resolution (43); whereas in the CVA airway, the violent, high-frequency mechanical impact of coughing breaches the physiological threshold, steering Piezo1 gating toward sustained Ca²^+^ overload and NLRP3 inflammasome activation. None of the above studies was conducted in a CVA model or human CVA samples. Extending these findings to the CVA airway is mechanistically plausible but remains an inference requiring direct validation in CVA-specific systems. A study conducted in a CVA animal model has confirmed that the NLRP3 inflammasome is significantly activated in CVA lung tissue and that NLRP3 inhibition ameliorates CVA-related pulmonary dysfunction (101). Although this study did not address the upstream Piezo1 link, it provides preliminary direct evidence of NLRP3–IL-1β axis involvement in CVA and, to some extent, narrows the above causal gap. With respect to CVA, the above evidence collectively supports a reasonable inference: in the high-mechanical-stress microenvironment created by repeated violent coughing, Piezo1 in alveolar, interstitial, or recruited macrophages may be activated according to their local mechanical environment and may participate in local inflammation through calcium-dependent downstream pathways. Future research may prioritize narrowing this evidence gap at three levels: validating the impact of myeloid-specific Piezo1 conditional knockout on airway inflammation and cough reflex in CVA animal models; detecting the correlation between Piezo1 expression and NLRP3 activation markers in macrophages from induced sputum or bronchoalveolar lavage fluid of CVA patients; and applying simulated cough mechanical stress (e.g., cyclic mechanical stretch) to human airway macrophages *in vitro* to directly observe the response of the Piezo1-NLRP3 axis.

Among the above trans-organ evidence, the report by Malko et al. (2023) presents a noteworthy contrast: in LPS-stimulated microglial cells, Piezo1 activation inhibits NF-κB nuclear translocation and reduces TNF-α/IL-6 production (58), exhibiting an anti-inflammatory effect opposite to the direction focused on in this review. As central nervous system–resident myeloid cells, microglia differ significantly from peripheral tissue-resident macrophages in their transcriptional programs, and Piezo1 downstream signaling coupling is not identical between the two. Malko et al. used Yoda1, a chemical agonist that produces sustained low-amplitude calcium signals; these signals inhibit NF-κB transcriptional activity via the calcineurin–NFAT pathway. In contrast, the mechanical stimulation produced by coughing has the characteristics of high amplitude, high frequency, and instantaneous burst, and the calcium transients it elicits differ fundamentally from those of Yoda1 in amplitude and kinetics: excessive calcium influx from intense mechanical stimulation exceeds cellular buffering capacity and preferentially drives NLRP3 inflammasome assembly and IL-1β maturation via potassium efflux (KCNN4) and mitochondrial calcium overload. The reason these two types of calcium signals activate fundamentally different downstream pathways lies in the fact that the frequency, amplitude, and duration of cytoplasmic calcium signals are themselves physical parameters encoding effector selection: sustained low-frequency calcium oscillations preferentially match the high-affinity calcium-sensing sites of the calcineurin–NFAT pathway, whereas high-frequency, high-amplitude mechanically gated calcium transients surpass this threshold to activate potassium channels (KCNN4) and the mitochondrial calcium uniporter (MCU), diverting the signal toward NLRP3 assembly. The study by Atcha et al. (2021) provides direct support for this framework: Piezo1-regulated macrophage calcium influx amplitude increases along a matrix stiffness gradient, and only large-amplitude calcium signals generated in high-stiffness environments couple with NF-κB activation and proinflammatory polarization (34). Applying the anti-inflammatory conclusions drawn from Yoda1 chemical agonism in microglia directly to airway macrophages subjected to high-frequency mechanical stress is not warranted. The fundamentally different downstream effects of Piezo1 under different conditions indicate that this channel is exquisitely regulated by the microenvironment to function as a signaling hub, with its net effect determined by the combined action of cell type, stimulus intensity, and microenvironmental context.

## 4 Calcium transients are amplified by calmodulin and the PI3K/AKT hub into a sustained proinflammatory program

Although the calcium influx triggered by Piezo1 opening is strong, it is also brief. To transform a millisecond ionic pulse into a sustained inflammatory program capable of remodeling the cellular phenotype, this signal must be precisely translated and amplified by the intracellular signaling network. The core of this translation is the molecular docking between calcium-sensing proteins and the lipid kinase network, together with the deep involvement of mitochondria as a calcium-buffering and signal-amplifying hub.

### 4.1 Free calcium is translated by calmodulin into a sustained and cascade-amplifiable protein phosphorylation signal

The free calcium ions that enter the cytoplasm first bind to the core calcium-sensing protein calmodulin (CaM), triggering helical rearrangement and conformational inversion of the latter and enabling it to bind and activate a variety of downstream effectors. Calmodulin thereby serves as a central node that converts a transient ionic signal into a durable biochemical signal. On the one hand, the complex formed by calcium and calmodulin can directly bind to the evolutionarily conserved motif of class III phosphoinositide 3-kinase, regulating its lipid kinase activity and transmitting the signal downstream (52); on the other hand, calcium and calmodulin can further amplify the signal through calmodulin-dependent protein kinase kinase and its downstream target protein kinases. In macrophages, Piezo1-mediated calcium influx has been shown to drive downstream transcriptional programs via calcium-induced calmodulin-dependent protein kinase II, thereby remodeling the metabolic and inflammatory state of the cell (41, 48). In addition to the kinase pathway, calcium influx can also activate the calcium-dependent protease pathway, constituting yet another calcium-dependent branch of macrophage Piezo1-induced inflammation (47). This process translates the inherently extremely brief calcium pulse produced by Piezo1 opening into a protein phosphorylation signal of longer duration that can be continuously amplified through enzymatic cascades, serving as the initiating hub for subsequent pathological responses.

### 4.2 Calcium signaling participates in dual-site phosphorylation of the PI3K/AKT cascade through the calmodulin-dependent kinase network

Among the downstream pathways activated by calmodulin, the PI3K/AKT cascade holds a pivotal position for the proinflammatory transcription and metabolic reprogramming of macrophages. Classic mechanistic studies show that calcium and calmodulin can, via calmodulin-dependent protein kinase kinase 2, directly phosphorylate protein kinase B at the dual sites Thr308 and Ser473, with potency comparable to the classic pathway mediated by phosphoinositide-dependent protein kinase 1, and that calcium chelation or calmodulin inhibition can markedly attenuate phosphorylation of protein kinase B (50). This coupling of calcium–calmodulin–PI3K/AKT is likewise validated in immune cells: in mast cells, calcium mobilization regulates, via calmodulin, the activation of PI3K-dependent downstream effectors and the phosphorylation of protein kinase B (51). The protein kinase B pathway integrates inflammatory and metabolic signals and determines the M1/M2 polarization fate of macrophages, and its activation broadly regulates proinflammatory cytokine production, phagocytosis, autophagy, and cellular metabolism (56). In the scenario directly driven by Piezo1, mechanically gated calcium influx can promote aerobic glycolysis in macrophages via the calmodulin-dependent protein kinase II–hypoxia-inducible factor-1α axis and enhance their proinflammatory cytokine secretion under lipopolysaccharide stimulation, thereby integrating mechanical signaling, metabolic reprogramming, and the proinflammatory program (41). PI3K/AKT regulation of macrophage polarization is context-dependent. In tissue-repair or inflammation-resolution microenvironments, activation of this pathway promotes M2 polarization and tissue repair (49), and matrix stiffness can also drive macrophage repolarization toward the M2 phenotype through the Piezo1–YAP signaling axis (35). The preferential pro-inflammatory rather than anti-inflammatory direction of PI3K/AKT in the CVA airway depends on the combinatorial features of upstream signal input. Different AKT isoforms have opposing effects on polarization direction—Akt1 biases toward pro-inflammatory transcription while Akt2 biases toward M2 polarization (119)—and the high-frequency calcium transients generated by cough mechanical force via Piezo1 persistently activate the CaMKII–HIF-1α axis, locking the output of this pathway onto the Akt1-dependent glycolytic-proinflammatory program. Downstream mTORC1 is likewise governed by upstream input: in an IL-4-dominated environment, mTORC1 drives fatty acid oxidation via IRF4 and PPARγ to support M2 polarization; in the CVA airway, however, the priming background maintained by baseline type-2 micro-inflammation and the Ca²^+^/CaM signals generated by sustained mechanical stress together bias mTORC1 metabolic output toward HIF-1α-dependent aerobic glycolysis. The persistent activation of NF-κB by Ca²^+^/calmodulin and the M2 transcription mediated by IL-4/STAT6 compete for shared co-activators; high-frequency mechanically gated calcium transients continuously tip the balance toward NF-κB, attenuating the anti-inflammatory output of STAT6 (120). In other tissue microenvironments lacking sustained mechanical stress input, the same pathway can still execute M2 polarization and tissue repair functions.

### 4.3 Mitochondrial calcium overload triggers a reactive oxygen species burst and drives macrophages into an irreversible proinflammatory activation state

During the large-scale calcium influx mediated by Piezo1, mitochondria—the primary intracellular center for calcium buffering—are deeply involved. Mitochondria take up excess cytoplasmic calcium in large amounts via the mitochondrial calcium uniporter in an attempt to maintain homeostasis; however, when the calcium load exceeds their buffering capacity, severe mitochondrial calcium overload ensues and triggers mitochondrial dysfunction. Studies confirm that calcium uptake mediated by the mitochondrial calcium uniporter is a key link in macrophage inflammation: the mitochondrial calcium overload it drives can directly promote NLRP3 inflammasome activation and IL-1β production, whereas knockout of this uniporter alleviates mitochondrial dysfunction and suppresses inflammation (53); this uniporter also enhances NLRP3 inflammasome activation through mechanisms such as promoting phagosomal membrane rupture (54). One direct consequence of mitochondrial calcium overload is depolarization of the mitochondrial membrane potential and abnormal opening of the mitochondrial permeability transition pore, which in turn impedes electron transport along the respiratory chain and leads to explosive generation of mitochondrial reactive oxygen species (mtROS). In macrophages, the production of mtROS, changes in membrane potential, and opening of the mitochondrial permeability transition pore have been confirmed to be tightly coupled with NLRP3 inflammasome activation and IL-1β release, and restoring mitochondrial function can markedly suppress this inflammatory process (55). At this point, the calcium signal triggered by Piezo1 pushes macrophages toward a proinflammatory threshold through two mutually reinforcing pathways: one is the proinflammatory transcription and metabolic reprogramming mediated by calcium–calmodulin–PI3K/AKT, and the other is the oxidative stress burst caused by mitochondrial calcium overload. The synergy of abnormal activation of the kinase network and mitochondrial oxidative stress provides the complete upstream conditions for the subsequent assembly and activation of the NLRP3 inflammasome, driving macrophages from a controllable stress state into an irreversible state of comprehensive inflammatory activation.

## 5 Mechanically related NLRP3 inflammasome assembly is an important link of innate immune amplification

The upstream PI3K/AKT signaling activation and the mtROS burst ultimately converge at the subcellular level to drive the assembly and activation of the molecular complex with the greatest amplifying effect in innate immunity—the NLRP3 inflammasome. Activation of the NLRP3 inflammasome follows a strict two-signal logic, and the airway microenvironment of CVA happens to simultaneously possess the conditions satisfying both signals, allowing mechanical stress to drive excessive inflammatory cytokine release via this amplification node.

### 5.1 Basal micro-inflammation and cough mechanical stress respectively provide the priming signal and the activation signal to complete NLRP3 two-signal licensing

Activation of the NLRP3 inflammasome requires two signals that are independent of each other in timing and nature. The first signal, the priming signal, is usually provided by pattern-recognition receptors or cytokine receptors that activate nuclear factor-κB and upregulate transcription of NLRP3 and pro-IL-1β; studies show that the NLRP3 expression level is itself a rate-limiting checkpoint for activation (61). In the CVA airway microenvironment, this priming signal is derived mainly from basal type-2 eosinophilic micro-inflammation and allergen exposure. The source of the priming signal may differ among patients with CVA. In eosinophilic CVA, type-2 cytokines—particularly IL-13 and IL-4—upregulate NLRP3 and pro-IL-1β transcription through STAT6 and NF-κB pathways; in non-eosinophilic or mixed-granulocytic phenotypes, epithelial-derived alarmins (IL-33 and TSLP) may provide an equivalent priming function through TLR/IL-1R signaling. Induced-sputum studies have revealed the inflammatory heterogeneity of CVA: in a study of 40 patients, the eosinophilic phenotype accounted for 32.5%, the paucigranulocytic phenotype for 52.5%, and the neutrophilic phenotype for 15%; a sputum neutrophil percentage above 46% predicted a poor response to anti-asthma treatment (14). This heterogeneity may contribute to variation in corticosteroid responsiveness. The NLRP3 inflammasome itself is also context-dependent: in experimental allergic asthma, NLRP3 deficiency paradoxically aggravated airway barrier injury and airway hyperresponsiveness (102), suggesting that NLRP3 may exert a protective effect under certain conditions. This review focuses on the proinflammatory pathological effects of repeated NLRP3 activation driven by high-frequency mechanical stress in CVA. Recent evidence also indicates that group 2 innate lymphoid cells (ILC2s) express Piezo1 and respond to mechanical cues (46), potentially helping maintain the type-2 inflammatory environment that provides NLRP3 priming in eosinophilic CVA.

The second signal, the activation signal, is provided by cellular perturbations such as potassium efflux, calcium dysregulation, and mtROS. Cough-generated mechanical stress is a potent source of these activation signals: shear stress alone is sufficient to serve as signal two in lipopolysaccharide-primed macrophages, inducing Piezo1-dependent ASC aggregate formation, gasdermin D cleavage, caspase-1 activation, and IL-1β secretion, with the process depending mainly on NLRP3 (73). Piezo1-mediated calcium influx activates calcium-activated potassium channels, drives potassium efflux, and directly promotes NLRP3 inflammasome activation, thereby lowering the activation threshold (17). Potassium efflux and calcium influx also act together to drive mtROS generation and trigger NLRP3 activation (65). Persistently activated PI3K/AKT signaling further impairs the clearance of damaged mitochondria by suppressing AMPK/mTOR-mediated autophagy (57, 62); the resulting accumulation of ROS-producing mitochondria is itself sufficient to activate the NLRP3 inflammasome (66). In the CVA airway, the priming signal and the activation signal are thus satisfied concurrently, allowing mechanical stress to complete the two-signal licensing of NLRP3.

### 5.2 The mechanical activation signal completes supramolecular assembly of the inflammasome through NEK7-mediated oligomerization and ASC aggregate assembly

After two-signal licensing, NLRP3 must undergo precise assembly from an inactive monomer into an active supramolecular complex. Potassium efflux is the key trigger of this assembly, and NIMA-related kinase 7 (NEK7) is the core mediator downstream of it. Studies establish that NEK7, as an NLRP3-binding protein, regulates the oligomerization and activation of NLRP3 downstream of potassium efflux; in the absence of NEK7, the NLRP3 activation signal cannot induce caspase-1 activation and IL-1β release, and the formation of high-molecular-weight NLRP3–NEK7 complexes and the oligomerization and aggregate formation of ASC in activated macrophages likewise disappear (63). Cryo-electron microscopy structures further reveal that NEK7 simultaneously engages the leucine-rich repeat and the NACHT domain of NLRP3 with its carboxy-terminal lobe and bridges adjacent NLRP3 subunits through a bipartite interaction, thereby licensing inflammasome assembly at the structural level (64). During assembly, the inactive NLRP3 cage-like structure is released and, with the assistance of NEK7, forms an NLRP3–NEK7–ASC disc; through homotypic PYD–PYD and CARD–CARD interactions, a filamentous scaffold is formed, ultimately recruiting and aggregating ASC into a micrometer-scale radial aggregate (60). This supramolecular complex then recruits the inactive caspase-1 precursor to complete the final assembly of the inflammasome (59). Notably, inflammasome assembly is accompanied by clear organelle relocalization: NLRP3, localized in the resting state to the endoplasmic reticulum structure, redistributes together with ASC toward the perinuclear space upon activation and colocalizes with organelle clusters of the endoplasmic reticulum and mitochondria, and this spatial rearrangement brings inflammasome assembly into physical proximity with mitochondria-derived reactive oxygen species signaling (66). At the whole-animal level, activation of macrophage Piezo1 has been confirmed to enhance inflammation via the NLRP3/nuclear factor-κB pathway, whereas its deletion suppresses NLRP3 expression and p65 nuclear translocation, providing *in vivo* causal evidence for the mechanical force–Piezo1–NLRP3 assembly chain (44).

### 5.3 Caspase-1 releases mature IL-1β through gasdermin D pore formation, provoking airway inflammation and laying the foundation for chronic feedback

The assembled NLRP3 inflammasome causes caspase-1 to undergo autocleavage and activation, and the activated caspase-1 then executes two decisive downstream reactions. First, it cleaves the IL-1β precursor and the IL-18 precursor to generate biologically active mature cytokines (59). Second, it cleaves gasdermin D, releasing its amino-terminal fragment with pore-forming activity; this fragment translocates to and oligomerizes at the plasma membrane to form nanometer-scale transmembrane pores, a process established as the executioner mechanism of pyroptosis (67). The specific recognition by caspase-1 of the gasdermin D cleavage site has been structurally resolved, and a specific peptide inhibitor has been developed accordingly, providing a molecular target for intervening in this link (68). The formation of gasdermin D pores has significance beyond cell death: it constitutes the direct conduit for mature IL-1β to be released extracellularly across the intact lipid bilayer. Crucially, macrophages can, in a surviving hyperactivated state, continuously secrete IL-1β through gasdermin D pores rather than necessarily proceed to lytic death (69); these pores can also mediate the release of soluble cytoplasmic proteins such as IL-1β and IL-18 without depending on cell lysis (70). This survival-secretion property has critical pathological implications: it enables airway macrophages subjected to repeated mechanical stimulation to continuously pump mature IL-1β into the airway microenvironment without being exhausted, establishing the cellular basis for a self-sustaining inflammatory feedback loop. Clinical-sample evidence provides direct support for the disease relevance of this mechanism: the expression of NLRP3, caspase-1, and IL-1β is markedly elevated in the airways of patients with asthma, including sputum macrophages (71), and sustained activation of the NLRP3 inflammasome is regarded as an important driver of airway inflammation and airway hyperresponsiveness in severe and refractory asthma, with administration of an NLRP3 inhibitor in animal models suppressing airway hyperresponsiveness and pulmonary inflammation (72). Notably, the NLRP3 inflammasome has likewise been confirmed to be significantly activated in a CVA animal model, and NLRP3 inhibition effectively ameliorates CVA-related pulmonary dysfunction (101), providing preliminary direct evidence for the involvement of this effector-terminal mechanism in CVA. Large amounts of mature IL-1β are discharged into the airway microenvironment through gasdermin D pores, thereby provoking tissue inflammation and transmitting the signal to the next link—the vagal sensory nerves innervating the airway. At this point, the candidate intracellular pathway from mechanical gating to inflammatory cytokine release is connected (**Figure 3**).

**Figure 3:**
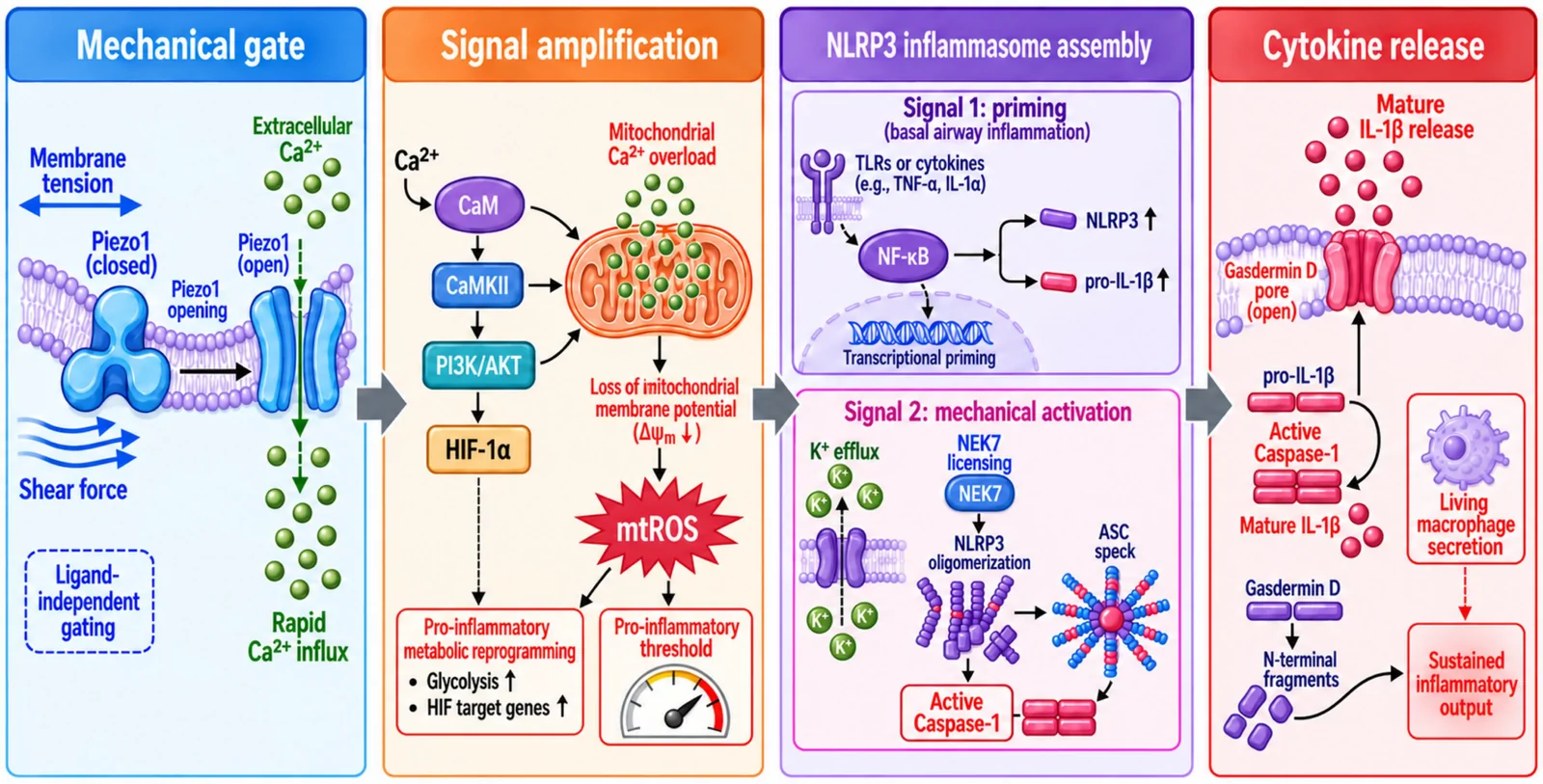
The Piezo1–Ca²^+^–PI3K/AKT–mtROS–NLRP3 mechanoinflammatory cascade in airway macrophages. The image presents four sequential and spatially independent modules illustrating the proposed conversion of mechanical stimuli into inflammatory cytokine release by airway macrophages. The Mechanical gate module shows membrane tension and shear force promoting the transition of Piezo1 from the closed to the open state, followed by rapid Ca²^+^ influx. The Signal amplification module shows Ca²^+^-dependent activation of CaM, CaMKII, and PI3K/AKT-related signaling, together with mitochondrial Ca²^+^ overload, loss of mitochondrial membrane potential, mtROS generation, and proinflammatory metabolic reprogramming. The NLRP3 inflammasome assembly module shows the contribution of a priming signal, mediated by basal airway inflammation and NF-κB activation, and a mechanical activation signal involving K^+^ efflux, NEK7-dependent NLRP3 oligomerization, ASC speck formation, and caspase-1 activation. The Cytokine release module shows caspase-1-mediated processing of pro-IL-1β and gasdermin D cleavage, followed by the release of mature IL-1β through gasdermin D pores while the macrophage remains viable. Arrows between the modules indicate the sequential progression of the proposed mechanism; the membrane and cytosolic structures shown in each module represent local views of the corresponding cellular processes.

## 6 Closure of the neuroimmune synapse may form a hypersensitive feedback loop from cough to inflammation and back to cough

The mature inflammatory cytokines released by macrophages form a high concentration proinflammatory microenvironment in the airway submucosa and may remodel the electrophysiological characteristics of the sensory nerves innervating this region. This link converts local innate immune activation into cough behavior that can be perceived by the patient and repeatedly triggered, and ultimately may allow the entire signaling axis to close into a self-reinforcing vicious cycle.

### 6.1 Macrophage-derived IL-1β may sensitize vagal C-fibers through IL-1R1-mediated neuroimmune signaling

Unmyelinated vagal C-fiber terminals are distributed within the lamina propria and epithelial compartments of the airway mucosa and constitute an important peripheral sensory component of the cough reflex (74, 83). Mature IL-1β released from macrophages through gasdermin D pores may diffuse through the local interstitium and bind to interleukin-1 receptor type 1 (IL-1R1) on vagal sensory terminals. A recent mouse study detected IL-1R1 on nodose ganglion sensory neuron cell bodies and along the vagus nerve by immunohistochemistry. *In vivo* calcium imaging further showed that direct application of IL-1β to the vagus nerve elicited cytokine-specific responses in a subset of nodose ganglion sensory neurons (82). These findings support the capacity of vagal sensory neurons to detect IL-1β through IL-1R1-related signaling, although the study was not conducted in airway-specific neurons or a model of cough-variant asthma.

The anatomical proximity between macrophage IL-1β release sites and vagal sensory terminals may permit efficient paracrine immune-to-neural communication (74). In this review, the term “functional neuroimmune synapse” refers to such anatomical proximity and functional coupling rather than to a classical synaptic structure with a defined synaptic cleft. Direct confirmation of this proposed coupling in the airways of patients with cough-variant asthma remains necessary. Airway neuroimmune regulation is also bidirectional, as activated sensory nerves can modulate local inflammation (84). Within the proposed pathogenic loop, macrophage-to-sensory-nerve signaling may contribute to the maintenance of cough hypersensitivity.

Airway mechanotransduction may also involve parallel cellular pathways. Cough-associated mechanical stress may promote epithelial perturbation and the release of alarmins, including ATP, TSLP, and IL-33, and may influence mechanically or chemically sensitive channels on sensory nerve terminals. Activated sensory nerves can release calcitonin gene-related peptide and tachykinins, including substance P, through local axon-reflex mechanisms, thereby contributing to neurogenic inflammation. These pathways may operate alongside the macrophage Piezo1–NLRP3 axis within the proposed mechanobiological network of cough-variant asthma.

### 6.2 IL-1β-related neuroimmune signaling and TRP-channel sensitization may jointly promote peripheral hypersensitivity

Evidence from studies of intracellular signaling in peripheral sensory neurons and cytokine sensing by vagal sensory neurons suggests that IL-1β–IL-1R1 signaling may contribute to neuronal activation and sensitization (81, 82). In peripheral sensory neurons, cytokine receptor signaling can engage intracellular pathways, including mitogen-activated protein kinase pathways, and modulate pronociceptive receptors and ion channels, thereby increasing neuronal sensitivity and excitability—a process defined as peripheral sensitization (81). However, a continuous molecular pathway directly linking IL-1R1 to TRPV1 or TRPA1 has not yet been established in airway sensory terminals in cough-variant asthma. At the molecular mechanistic level, TRPV1 can be sensitized either directly through phosphorylation by protein kinase C or indirectly by inflammatory mediators such as prostaglandin E2, bradykinin, and nerve growth factor via their respective receptors; multiple sensitization modes superimpose on one another, finely upregulating channel activity, increasing the open probability, and markedly lowering its activation threshold (77). Chronic airway inflammation can also induce marked overexpression of TRPV1 in pulmonary sensory neurons, placing the nerve terminals in an irritable state (78); in the bronchial tissue of patients with chronic cough, a marked increase in TRPV1-immunoreactive nerves is indeed observed, and inflammatory mediators can upregulate their cough sensitivity (80). In parallel with TRPV1, the TRPA1 channel is highly sensitive to cold air, environmental irritants, and the reactive electrophilic molecules generated during oxidative stress and inflammation, and its activation can excite vagal bronchopulmonary C-fibers and provoke cough, constituting another important sensitizing amplification pathway (79). These mechanisms collectively render airway vagal nerve terminals extremely hyperexcitable, and the cough reflex threshold drops sharply.

### 6.3 Hypersensitive vagal terminals amplify minor stimuli into high-frequency discharges transmitted to the cough center and ultimately anchor the physical–immune–neural vicious cycle

The sensitized vagal nerve terminals can amplify minor physiological stimuli originally insufficient to elicit cough—such as the cold-air convection produced by breathing, slight mucus disturbance, and even speaking and deep breathing—into dense, high-frequency action potentials, which are transmitted via vagal afferent fibers to the cough center in the nucleus tractus solitarius of the brainstem for integration, thereby triggering paroxysmal spasmodic dry cough that the patient finds difficult to suppress (83). This heightened excitability, in which both peripheral and central levels participate, is precisely the neurobiological substance of cough hypersensitivity syndrome (75, 76), and it corroborates the defining feature emphasized by cough hypersensitivity syndrome that excessive cough is produced in response to low-level mechanical stimulation (11). At this point, the closing logic of the entire signaling axis can be summarized as follows: each round of violent cough triggered by amplification through hypersensitive nerves re-creates high-velocity airflow shear stress and severe wall deformation locally in the airway, again physically opening the Piezo1 channels on the surface of local macrophages, restarting the Piezo1–Ca²^+^–PI3K/AKT–NLRP3–IL-1β proinflammatory cascade, and thereby maintaining the hypersensitive sensitization of the vagal nerves. The pathological closed loop in which coughing initiates inflammation and inflammation, in turn, reinforces coughing may thereby be maintained. This closed loop is not a purely functional cycle but is accompanied by definite structural remodeling: human airway biopsy evidence shows that the sensory nerve density and branching of the airway epithelium are markedly increased in patients with chronic cough, indicating that sensory nerve plasticity participates in the formation and maintenance of cough hypersensitivity and provides an anatomical material basis for the vicious cycle (85). Meanwhile, the activated sensory nerves, through efferent release of neuropeptides such as calcitonin gene-related peptide and tachykinins, can further aggravate local neurogenic inflammation, constituting yet another positive feedback on this closed loop (86). Taken together, the establishment and consolidation of the mechanical force–Piezo1–inflammasome–vagal nerve closed loop mechanistically explain why CVA manifests as an intractable, protracted chronic dry cough readily provoked by minor stimuli. In this process, central sensitization represents another pivotal mechanism reinforcing cough hypersensitivity. Dense high-frequency discharges from peripheral vagal C-fibers and macrophage-derived IL-1β act on the brainstem nucleus tractus solitarius (NTS) via axonal or humoral pathways, inducing glutamatergic synaptic facilitation and microglial neuroinflammation in the NTS; At the molecular level, IL-1β enhances NMDA receptor phosphorylation via IL-1R1 on NTS neuron surfaces, increasing excitatory synaptic transmission efficacy while directly suppressing GABA and glycine receptor-mediated inhibitory currents, shifting the excitation-inhibition balance of the cough center network toward excitation (75, 76). Activated NTS microglia further release proinflammatory mediators including BDNF and TNF-α: BDNF acts via TrkB receptors to downregulate postsynaptic KCC2 expression, impairing chloride extrusion capacity and converting GABAergic currents from hyperpolarizing to depolarizing, effectively reversing inhibitory synapses into excitatory ones (75); TNF-α promotes AMPA receptor insertion into the synaptic membrane and enhances NMDA-mediated currents (76). These molecular events collectively lead to a persistently lowered amplification threshold for cough signaling at the brainstem level; even when peripheral proinflammatory input diminishes, the established synaptic potentiation can maintain excessive cough reflex activity (82, 84). Concurrently, descending inhibitory control from higher cortical and limbic networks is diminished, leading to disinhibition of central cough-gating circuits (82, 84). The establishment of central sensitization endows the cough reflex with self-perpetuating properties. This clinically implies that in refractory CVA where NTS and cortical plasticity are established, targeting peripheral mechanoinflammation alone may be insufficient to fully abolish cough, highlighting the therapeutic rationale for combining peripheral and central neuromodulatory strategies.

## 7 Targeting intrinsic mechanical inflammation provides a new dimension of molecular intervention for refractory cough-variant asthma

An in-depth understanding of the mechanism of mechano-immune conversion not only reconstructs the pathogenic map of CVA but also points directly to a series of new therapeutic targets. The mechanical-stress–related signaling axis may form signal-bypass resistance to transcription-dependent corticosteroids, and targeted interventions along the physical source, signal hub, and effector terminal levels have shown feasibility in breaking this cascade in preclinical models. All of the above intervention strategies are supported by preclinical experimental evidence, and their efficacy and safety in CVA patients require validation in subsequent disease-specific trials.

### 7.1 The mechanical-stress–related signaling axis may form signal-bypass resistance to transcription-dependent corticosteroids

Clinically, a considerable proportion of patients with CVA respond poorly to conventional inhaled corticosteroids, and those with a longer chronic course have worse efficacy (7). It is important to note that steroid resistance in clinical practice is multifactorial. Factors such as incorrect inhaler use, poor adherence, diagnostic overlap with other cough conditions (e.g., UACS, GERD), and established airway remodeling all contribute to poor treatment responses. The mechanoinflammatory axis proposed here represents one additional, and previously overlooked, pathway that may operate in a subset of patients. Inhaled corticosteroids act primarily through glucocorticoid receptor–mediated transcriptional suppression, targeting the chronic inflammatory process that depends on gene transcription, exemplified by type 2 inflammation. However, the mechanical-stress-driven Piezo1–Ca²^+^–PI3K/AKT–NLRP3 axis is characterized at its upstream links by rapid transmembrane ionic flux and a kinase phosphorylation cascade; these processes occur at the post-translational and ionic-flux levels and do not directly depend on classic transcriptional regulation, and therefore have intrinsic signal-bypass resistance to corticosteroids that act mainly on transcription. This analysis is consistent with known mechanisms of corticosteroid resistance: oxidative stress lowers the activity and expression of histone deacetylase 2 via the phosphoinositide 3-kinase δ pathway, considered one of the core mechanisms of corticosteroid resistance in severe asthma, chronic obstructive pulmonary disease, and smoking-related asthma (98). Consistently, non-type-2 airway inflammation dominated by neutrophils and driven by interleukin-17 and macrophage-derived TNF-α is generally insensitive to corticosteroids (99), and sustained activation of the NLRP3 inflammasome is precisely an important driver of airway inflammation in severe, refractory asthma (72). In experimental models of steroid-resistant asthma, excessive NLRP3/IL-1β activation has been directly shown to drive the steroid-resistant phenotype, with NLRP3 and IL-1β expression levels positively correlated with sputum neutrophilia and asthma loss of control; NLRP3 inhibition can reverse the insensitivity of neutrophilic airway inflammation to corticosteroids (106). This suggests that corticosteroids alone may be insufficient to fully interrupt a physical inflammatory process driven by ionic flux and a kinase cascade. The Piezo1–NLRP3 axis provides a candidate mechanistic explanation for a subset of CVA patients with poor corticosteroid responses.

### 7.2 Physical-source blockade: cutting off the proinflammatory input at the most upstream of mechanotransduction with Piezo1 modulators such as GsMTx4

Since the conversion of cough mechanical stress into calcium signaling via Piezo1 is the potential upstream trigger of the entire proinflammatory cascade, blockade at this most upstream link theoretically has the interventional value of cutting off the proinflammatory input at its source. The spider-toxin-derived peptide GsMTx4 provides a proof of concept for this. Studies confirm that GsMTx4 can reversibly inhibit the mechanically induced current of Piezo1 at micromolar concentrations with an affinity of approximately 155 nanomolar, markedly right-shifting its pressure–gating curve, and acts as a gating modifier on the closed-state channel while having no apparent effect on non-mechanosensitive channels (88). Its mechanism of action is distinctive: GsMTx4 does not directly block the pore but inserts into the near-aqueous interface of the membrane lipid, acting as an area reservoir that penetrates deeper into the bilayer when the membrane is under tension, thereby locally reducing the effective membrane tension acting on channel gating (87). This membrane-tension-based source blockade strategy can intercept the conversion of mechanical stress into calcium influx at its very first link, cutting off the force-to-signal conversion at the biophysical level. More broadly, owing to the resolution of cryo-electron microscopy structures and the various agonists and inhibitors obtained through high-throughput screening, Piezo1 has been regarded as a druggable target with clear potential, and small-molecule modulators targeting its mechanical gating properties are under active development (89, 90). Delivering such Piezo1 modulators to the airway locally via the inhaled route may simultaneously modulate mechanosensing by candidate lung macrophages and other airway cells, and its translational value depends on local delivery efficiency and cell selectivity. It therefore has unique value in the early intervention of refractory CVA. As a peptide, GsMTx4 faces barriers to inhaled delivery, including low bioavailability, potential immunogenicity, and insufficient cell-targeting specificity; these limitations are analyzed in detail at the end of Section 7.3.

### 7.3 Precise blockade of the signaling hub and the effector terminal synergistically shuts down the physical inflammatory cascade and relieves neural hypersensitivity

Beyond source blockade, precise intervention targeting the central amplification link and the inflammatory cytokine output terminal of the signaling axis can form a multi-level combined blockade with the upstream strategy. At the level of the intracellular signaling hub, phosphoinositide 3-kinase is the key node connecting calcium transients to the whole-cell inflammatory cascade, and developing an inhaled inhibitor for the airway locally is expected to block this link efficiently while minimizing systemic exposure. A nebulized phosphoinositide 3-kinase γ/δ inhibitor achieved a dosing profile of high intrapulmonary concentration and low systemic exposure in asthma and chronic obstructive pulmonary disease models, not only markedly attenuating eosinophilic and neutrophilic inflammation and reducing airway hyperresponsiveness, but also reversing smoke-induced corticosteroid-resistant pulmonary inflammation, providing direct preclinical evidence for local PI3K inhibition (96). At the inflammasome effector level, the highly selective small-molecule inhibitor MCC950 can directly bind the Walker B motif within the NACHT domain of NLRP3 and block ATP hydrolysis, thereby precisely locking wild-type NLRP3 and preventing its oligomerization (91, 92). This targeted intervention has shown definite efficacy in a variety of airway inflammation models: MCC950 can attenuate house dust mite-induced airway inflammation (93), markedly alleviate airway hyperresponsiveness, airway inflammation, and airway remodeling in a toluene diisocyanate-induced neutrophilic asthma model (94) and reduce pulmonary immunopathology in early respiratory syncytial virus infection, lowering the risk of long-term airway disease and childhood asthma (95). At the most distal cytokine level, blocking IL-1β signaling is also feasible: human trials demonstrate that an interleukin-1 receptor antagonist can markedly reduce airway neutrophilic inflammation induced by inhaled endotoxin and downregulate related proinflammatory factors, with good safety (97). It should be emphasized that the significance of these targeted approaches lies not only in directly suppressing inflammation but, more importantly, in cutting off the key nodes of the vicious cycle—by rapidly reducing the source of the proinflammatory factor mature IL-1β, the sustained sensitization of vagal sensory nerves can be relieved, thereby lowering the hypersensitive state of the cough reflex and creating conditions for breaking the cough–inflammation–cough closed loop. All of the above intervention strategies have shown preclinical efficacy, yet their translation to clinical application requires overcoming specific pharmacological and delivery limitations. The inhaled PI3Kγ/δ inhibitor has achieved a local dosing profile of high pulmonary concentration and low systemic exposure (96). Because PI3Kγ and PI3Kδ participate in leukocyte recruitment and immune-cell function, prolonged pulmonary inhibition requires direct evaluation of host-defense effects and definition of a therapeutic window in CVA. MCC950 has shown efficacy in multiple airway-inflammation models, but it remains a preclinical tool rather than an established therapeutic option; current translational reviews emphasize safer upstream NLRP3 inhibition, biomarker-guided patient selection, and localized delivery as future directions (105). Regarding IL-1 blockade, a human inhaled-endotoxin study showed that IL-1 receptor antagonism can reduce airway neutrophilic inflammation (97). Its efficacy, delivery strategy, and local safety in CVA require disease-specific clinical evaluation. GsMTx4, as a 34-amino-acid spider toxin-derived peptide, faces barriers via the inhalation route including accelerated mucociliary clearance, protease degradation preventing local concentrations from being maintained above the 155 nM inhibitory threshold, potential immunogenicity of exogenous peptides, and insufficient cell selectivity due to the widespread expression of Piezo1 (epithelial cells, smooth muscle cells, and endothelial cells all depend on its physiological functions) (88). Published pharmacokinetic data are limited to subcutaneous injection in mice (0.1–5 μM in skeletal muscle and heart at 24 h after 50 mg/kg dosing), with inhaled-route pharmacokinetic data entirely lacking. GsMTx4 validates the feasibility of pharmacologically blocking Piezo1 at the mechanistic level; its clinical translation via inhalation is limited by peptide bioavailability, immunogenicity, and insufficient cell selectivity. Developing small-molecule Piezo1 modulators with optimized aerodynamic particle size for airway deposition, protease resistance, and macrophage-targeted specificity is the priority direction for future translation (87). From a more macroscopic perspective, the above strategies collectively embody an emerging therapeutic paradigm, namely manipulating disease outcomes by regulating the mechanical properties of tissues and immune cells and their mechanosensing pathways, and mechano-immunology is thereby moving from concept toward interventionable clinical reality (100).

## 8 Discussion and perspectives

### 8.1 Current trends: the convergence of mechano-immunology and inflammasome research is reshaping the understanding of chronic airway diseases

The rapid development of innate immune mechanobiology has been systematically reshaping our understanding of inflammation. Mechanical force is no longer regarded as a mere physical background but is considered an immunoregulatory input equally important as chemical signaling: mechanosensitive channels such as Piezo1, as integrative hubs of mechano-immune signaling, exert gatekeeping functions in inflammation, pain, adaptive immunity, and neuroimmune interaction (18, 45). Two main lines of this understanding are converging. First, Piezo1 has been shown to be the core molecule by which innate immune cells sense intrapulmonary pressure fluctuations and initiate inflammation, and knockout of this channel in innate immune cells abolishes pulmonary inflammation (16). Second, the molecular bridge between mechanical force and the classic inflammasome has been progressively revealed: mechanical gating directly drives NLRP3 activation through ionic flux, and shear stress alone is sufficient to serve as its second signal (17, 73). This review is precisely grounded at this convergence point, connecting coughing—a macroscopic physical event occupying a central position in CVA—with microscopic airway inflammation through the continuous signaling axis of Piezo1–Ca²^+^–PI3K/AKT–NLRP3–vagal nerve. This framework provides an internally consistent mechanistic explanation for several clinical features long unexplained by the chemically centered paradigm—including the provocation of violent dry cough by minor mechanical stimuli, the poor response of some patients to corticosteroids, and the driving of airway remodeling by long-term cough—and assigns a concrete molecular substance to the long-ambiguous mechanical stimulation in cough hypersensitivity syndrome. Physical mechanical forces and chemical signals do not operate in isolation; rather, they undergo complex spatiotemporal integration at subcellular and tissue levels. Chemical mediators (such as allergen-induced type 2 cytokines) provide a persistent transcriptional background, whereas high-frequency cough mechanical forces deliver transient burst biochemical impulses via millisecond ion fluxes. This physical-chemical synergy redefines the maintenance mechanism of chronic airway inflammation. The substance of this synergy is a cross-time-scale superposition amplification process: within macrophages, the transcriptional program maintained by IL-13 and IL-4 via STAT6—including sustained upregulation of NLRP3 and pro-IL-1β—provides the requisite molecular substrates for inflammasome assembly on an hours-to-days timescale; cough-driven Piezo1–Ca²^+^ pulses superimposed on this transcriptional background at the millisecond timescale provide the “signal two” needed to trigger assembly. Chemical signaling alone is insufficient to activate NLRP3 (lacking signal-two inputs such as potassium efflux and mitochondrial calcium overload), and mechanical signaling alone is likewise insufficient (lacking transcriptional priming of NLRP3 protein); the spatiotemporal convergence of both in the same airway macrophage enables the cell to maintain persistent inflammasome activation even in the absence of ongoing antigen exposure. This cross-time-scale superposition model provides a mechanistic explanation for why CVA patients exhibit persistent airway inflammation even after withdrawal from acute allergen exposure.

### 8.2 Current bottlenecks: *in situ* dynamic capture of mechanical signals and the establishment of CVA-specific direct evidence remain core challenges

The explanatory power of the above framework is limited by current evidence gaps. Existing CVA studies have not yet resolved the spatial distribution of Piezo1–NLRP3 activation across different airway segments or among alveolar, interstitial, and recruited macrophages. First, a substantial portion of the mechanistic evidence integrated in this review comes from studies of macrophage Piezo1 in other organ systems such as liver fibrosis, inflammatory bowel disease, and acute kidney injury (42, 44, 47), as well as from ex vivo cells and generic inflammation models; extending this evidence to the CVA airway microenvironment currently lacks direct validation in disease-specific models. Second, the relationship between Piezo1 and downstream signaling has clear cell-type and context dependence, and in certain backgrounds its effect is even opposite to the proinflammatory direction (49, 58), and it can also promote inflammation resolution by enhancing efferocytosis (43); this suggests that the net effect of Piezo1 depends on the specific cellular state, activation intensity, and microenvironmental background, so simply equating it with being proinflammatory is not rigorous, and the specific context of CVA airway macrophages still requires precise definition. As detailed in Section 3.4, the anti-inflammatory feedback of Piezo1 observed in microglia via NF-κB inhibition (58) highlights that conclusions from general models cannot be directly extrapolated to the airway, necessitating precise delineation in CVA-specific macrophages. NLRP3 itself can also exert protective functions under certain conditions: in experimental allergic asthma, NLRP3 deficiency paradoxically aggravates airway barrier damage and airway hyperresponsiveness (102), indicating that the “context-dependence” principle extends to the NLRP3 effector terminal as well. A study in a CVA animal model has confirmed that the NLRP3 inflammasome is significantly activated in CVA lung tissue, and that NLRP3 inhibition ameliorates pulmonary dysfunction (101), providing preliminary disease-specific evidence for the downstream effector terminal, although the upstream Piezo1 mechanotransduction link still lacks direct validation in CVA. The above contradictory evidence defines the applicability boundary of this framework. These seemingly opposite conclusions can be understood within a unified “stimulus parameter–cell context” analytical framework: the net effects of Piezo1 and NLRP3 depend on the combination of stimulus intensity, duration, frequency, and cell lineage (see Section 2.3 threshold discussion and Section 3.4 Malko paradox analysis). The anti-inflammatory conclusions from Yoda1 chemical agonism in microglia are not contradictory to the response pattern of airway macrophages under intense mechanical stimulation, because the calcium signal amplitude, frequency, and downstream coupling pathways are fundamentally different. The aggravated phenotype observed upon NLRP3 deficiency in experimental allergic asthma reflects the homeostatic protective function of NLRP3 in the absence of sustained high-frequency mechanical stress, which is distinct from the proinflammatory pathological effects of NLRP3 after persistent activation driven by repeated violent coughing in the CVA airway. The core question revealed by contradictory evidence is: under what conditions does the Piezo1–NLRP3 axis transition from protective to pathogenic? This review, by defining the mechanical threshold and cellular context parameters for this transition, provides an analytical framework for unifying the above contradictions. Third, and most fundamentally, the challenge lies in the *in situ* dynamic capture of physical signals. The gating of Piezo1 occurs on a millisecond scale and is directly driven by membrane tension (39), whereas coughing is a transient, violent event difficult to reproduce without disrupting the three-dimensional mechanical environment of the airway; how to track in real time, at the *in vivo* or organoid level, the membrane tension changes, calcium transients, and inflammasome assembly within airway macrophages at the moment of violent coughing currently still lacks mature technical means. With the aid of genetically encoded calcium reporter systems, calcium influx has been attributable to Piezo1 in macrophages (34) but integrating such real-time reporters with controllable airway mechanical stimulation *in vivo* remains a methodological bottleneck urgently awaiting breakthrough in this field. In summary, the three major bottlenecks—(1) the persisting “causal gap” between trans-organ evidence and CVA-specific validation (as detailed in Section 3.4), (2) the context-dependent duality of Piezo1 signaling, and (3) the absence of real-time *in situ* imaging technology that preserves the 3D airway mechanical microenvironment—collectively define the boundary of current knowledge and chart the agenda for future research.

### 8.3 Future directions: translational advancement from *in vivo* mechanical imaging to Piezo1/NLRP3-targeted inhaled drugs

On the basis of the above bottlenecks, future research can proceed progressively at three levels. At the level of mechanistic validation, priority should be given to establishing disease-specific models of CVA, combining airway macrophage lineage tracing with myeloid-conditional Piezo1 knockout, simultaneously labeling alveolar, interstitial, and recruited macrophages and recording the anatomical compartments corresponding to induced sputum, bronchoalveolar lavage fluid, and lung tissue, to directly test the causal status and the direction of the net effect of the mechanical force–Piezo1–NLRP3 axis in this disease. At the level of technical methodology, it is urgent to integrate advanced membrane-tension probes, genetically encoded calcium and inflammasome-activity reporter systems, and *in vivo* multiphoton microscopy imaging to capture in real time and dynamically, while preserving as much as possible the three-dimensional physical environment of the respiratory tract, the full landscape of mechano-signal conversion within different candidate lung macrophage subsets at the moment of violent coughing, thereby transforming the currently indirect-evidence-based signaling chain into a directly observable process. At the level of translational medicine, the development and evaluation of three classes of interventions should be accelerated: first, Piezo1 modulators targeting the upstream of mechanotransduction, to block the mechanoinflammatory input at its source (89, 90); second, inhaled PI3K inhibitors targeting the intracellular amplification link, to achieve efficient local pulmonary blockade with low systemic exposure (96); and third, highly selective NLRP3 inhibitors and interleukin-1-blocking drugs targeting the inflammasome effector end, to cut off the source of proinflammatory factors and relieve neural hypersensitivity (91, 97). At the same time, developing biomarkers and phenotyping tools capable of reflecting mechanical inflammatory activity is expected to identify the patient subgroup dominated by mechanoinflammation and poorly responsive to conventional corticosteroids, thereby advancing individualized, stratified precision intervention. The deep integration of biomechanics and molecular immunology is expected to lead the development of the next generation of targeted therapies for refractory chronic cough and airway diseases. Future research should focus on three paths: first, establishing a CVA model with myeloid-specific Piezo1 knockout to verify the causal status of this signaling axis *in vivo*; second, developing *in vivo* multiphoton membrane-tension probes and real-time calcium-tracking systems to overcome the methodological bottleneck of capturing physical signals dynamically *in situ*; and third, evaluating the safety barriers of targeted interventions, with particular attention to the inhalation barriers of peptide modulators such as GsMTx4, the hepatotoxicity risk of MCC950-class molecules, and the immunosuppressive effects of inhaled PI3K inhibitors on pulmonary host defense, thereby advancing the development of safer next-generation targeted inhaled drugs.

Specifically, we propose a four-level concrete research plan: (1) Disease-specific *in vivo* validation: generating a myeloid-specific Piezo1 knockout crossed with a CVA-relevant mouse model (e.g., ovalbumin sensitization combined with chronic cough induction), with readouts including BALF IL-1β levels, vagal C-fiber electrophysiology, and citric acid–induced cough frequency, to directly test the causal role of macrophage Piezo1 in airway hyperresponsiveness and neural sensitization; (2) Real-time mechanotransduction imaging: developing intravital multiphoton platforms combining membrane-tension probes (e.g., Flipper-TR) with genetically encoded calcium indicators (e.g., GCaMP) in ex vivo precision-cut lung slices, applying calibrated pressure pulses mimicking cough-scale shear to track macrophage Piezo1 gating kinetics *in situ*; (3) Translational drug development: prioritizing small-molecule Piezo1 modulators with optimized inhalation pharmacokinetics (e.g., appropriate aerodynamic particle size for distal airway deposition, resistance to mucociliary clearance) over peptide-based compounds (such as GsMTx4) that face bioavailability and immunogenicity barriers; and (4) Biomarker-guided patient stratification: validating candidate biomarkers—including sputum or BAL fluid IL-1β levels, Piezo1/NEK7 qPCR expression in sputum or bronchoalveolar lavage fluid cells, and circulating gasdermin D fragments—in prospective CVA cohorts to identify the patient subset most likely to benefit from mechanoinflammation-targeted therapies.

Translating the concept of mechanoinflammation into clinical practice requires biomarkers that reflect the activity of the Piezo1–NLRP3 axis. Candidate markers include: (1) IL-1β levels in sputum or bronchoalveolar lavage fluid; (2) NLRP3 or caspase-1 in sputum or bronchoalveolar lavage fluid extracellular vesicles; (3) Piezo1 or NEK7 expression in sputum cells measured by qPCR; and (4) gasdermin D fragment levels in the circulation or sputum. Validating these markers in CVA cohorts would help stratify the patients most likely to benefit from mechanoinflammation-targeted therapy.

## 9 Conclusion

From the intersection of biophysics and molecular immunology, this review reconstructs the pathogenic map of CVA and proposes a paradigm shift from chemical immunity toward mechanoinflammation. Its central hypothesis is that CVA may simultaneously involve a microenvironmental homeostatic imbalance driven by airflow physical shear force and wall deformation, complementing the established paradigm centered on antigen-antibody chemical confrontation at the airway mucosa. Alveolar, interstitial, and recruited macrophages may constitute the candidate cellular basis of airway mechanotransduction according to their respective niches. These cells may use surface Piezo1 channels as sensing devices to translate cough-generated macroscopic mechanical stress into intracellular calcium transients; through amplification by the calmodulin and PI3K/AKT signaling hub and the synergy of mitochondrial calcium overload and reactive oxygen species bursts, they license NLRP3 inflammasome assembly and continuously release mature IL-1β via gasdermin D pores. The latter may sensitize vagal sensory nerves innervating the airway, sharply lowering the cough reflex threshold and may close a physical–immune–neural vicious cycle. This signaling axis assigns a molecular substrate to the mechanical stimulation in cough hypersensitivity syndrome and provides a mechanistic explanation for corticosteroid resistance and cough-driven airway remodeling. Beyond the chemical-immunity framework, incorporating mechanical force as an important driving dimension of airway inflammation helps more completely understand and intervene in this intractable disease.
